# Acknowledging the peer reviewers of *Journal of Medical Radiation Sciences*, October 2023–September 2024

**DOI:** 10.1002/jmrs.838

**Published:** 2024-12-03

The editorial review board, the Australian Society of Medical Imaging and Radiation Therapy (ASMIRT) and the New Zealand Institute of Medical Radiation Technology (NZIMRT) acknowledge the following peer reviewers for their dedication and commitment to the journal. Thank you for sharing your valuable time and knowledge.

## Outstanding Reviewers (Contributed Five Reviews or More)

James Stanley

Peter Kench

## The Following Reviewers Contributed Four Reviews

Amy Brown

Karen Dobeli

Georgia Halkett

Andrew Kilgour

## The Following Reviewers Contributed Three Reviews

Scott Jones

Chandra Makanjee

Martin Mitchell

Robba Rai

John Robinson

Clare Singh

Adam Steward

Francis Zarb

## The Following Reviewers Contributed Two Reviews

Dana Al‐Mousa

Nigel Anderson

Marilyn Baird

Rachael Beldham‐Collins

Pippa Bresser

Mikaela Dell'Oro

Victoria Earl

Ernest Ekpo

Sheryl Foster

Ziba Gandomkar

David Gilmore

Therese Gunn

Peter Hanna

Richard W. Harbron

James Hayes

Lynne Hazell

Bronwyn Hilder

Peter Hogg

Yobelli Jimenez

Gerhardus Koch

Gordon Mander

Kristie Matthews

Sibusiso Mdletshe

Martin Necas

Michael Neep

Jodie Nixon

Tracey Pieterse

William Rae

Oona Reardon

Meegan Shepherd

Zhonghua Sun

John Thompson

Kenton Thompson

Adrienne Young

Sarah Lewis

## The Following Reviewers Contributed One Review

Kamarul Abdullah

Laura Adamson

Verity Ahern

Theophilus Akudjedu

Sophie Alexander

Tonima Ali

Haney Alsleem

Sally Ball

Luke Barclay

Stevens Barry

Salvatore Berlangieri

Eva Bezak

Anita Bowman

Robyn Brady

Vicki Braithwaite

Maura Brown

Rachel Burton

Gemma Busuttil

Ethan Butson

Ke Cao

Yasmin Casmod

Chelsea Castillo

Crispen Chamunyonga

Shayne Chau

Nahid Chegeni

Deanne Chester

Kabilan Chokkappan

Jillian Clarke

James Crowhurst

Jennifer Dang

Rob Davidson

Jenna Dean

Pradip Deb

Edel Doyle

Elisabeth Elder

Kirsten Elleray

Doaa Elwadia

Andrew England

Brendan Erskine

Hafsa Essop

Mel Evans

Andrew Firman

Alannah Flockton

Janniko Georgiadis

Nicola Giannotti

Gopinath Gnanasegaran

Frances Grey

Roshini Gunewardena

Catriona Hargrave

Patrick Horsley

Chamandra Kammies

Abel Karera

Alannah Kejda

Toni Kelly

Ben Kennedy

Thandokuhle Khoza

Scott King

Tracy Kirkbride

Caroline Landelle

Drew Latty

Andrew Le

Fiona Lee

Margot Lehman

Shantel Lewis

Gaorui Liu

Kelly Lloyd

Magdalena Lutaka

Vanathy Manivasahan

Sonyia McFadden

Lisa McGuire

Glenda McLean

Vaughan Moutrie

Andrew Murphy

Soma Nesan

Michelle O'Connor

Katrina O'Keefe

Peter O'Reilly

Craig Opie

Brooke Osborne

Sharon Oultram

Vanessa Panettieri

Eric Pei Ping Pang

George Papadatos

Jennifer Parkes

Malene Pedersen

Melanie Penfold

Scott Penfold

Yosefa Pessin

Angelina Piccolo

Jo‐Anne Pinson

Natalie Pollard

Jonathan Loui Portelli

Suresh Rana

Mohammad Rawashdeh

Tristan Reddan

Majella Russo

Susan Said

Justin Newton Scanlan

Karen M. Scott

Anna Seeley

Michala Short

Kelly Smart

Kate Stewart

Laura Stork

Hironori Takahashi

Linda Thebridge

Samantha Thomas

Phuong Dung (Yun) Trieu

Wan Nordiana Wan Abdul Rahman

Yu‐Feng Wang

Abel Zhou

Angela Ziebell

## Peer Reviewer Recognition

JMRS reviewers can opt in to have their peer review contribution automatically added on Web of Science Reviewer Recognition Services (formerly Publons).

For more information please go to, https://authorservices.wiley.com/Reviewers/journal-reviewers/recognition-for-reviewers/publons.html

## Peer Reviewer Resources

Are you new to conducting and writing peer reviews? Check out these free peer reviewer guidelines and free self‐directed learning modules.

Wiley Journal Reviewers

https://authorservices.wiley.com/Reviewers/journal-reviewers/index.html

Wiley Researcher Academy: All you need to know to become an effective peer reviewer

https://www.wileyresearcheracademy.com/p/all-you-need-to-know-to-become-an-effective-peer-reviewer

Web of Science Academy

https://clarivate.com/webofsciencegroup/solutions/web-of-science-academy/

